# The Incidence of Chiari Malformations in Patients with Isolated Sagittal Synostosis

**DOI:** 10.1097/GOX.0000000000002090

**Published:** 2019-02-12

**Authors:** Amani Ali Davis, Giulio Zuccoli, Mostafa M. Haredy, Lauren Runkel, Joseph Losee, Ian F. Pollack, Mandeep S. Tamber, Elizabeth Tyler-Kabara, Jesse A. Goldstein, Ken-K Nischal

**Affiliations:** From the * Department of Ophthalmology, Children’s Hospital of Pittsburgh of UPMC, Pittsburgh, Pennsylvania; † Department of Radiology, Children’s Hospital of Pittsburgh of UPMC, Pittsburgh, Pennsylvania; ‡ Department of Plastic Surgery, Children’s Hospital of Pittsburgh of UPMC, Pittsburgh, Pennsylvania; § Plastic Surgery Department - Cleft and Craniofacial Unit, Sohag University Hospital, Sohag, 82524, Egypt; ¶ Department of Neurosurgery, Children’s Hospital of Pittsburgh of UPMC, Pittsburgh, Pennsylvania; ‖ UBC Department of Surgery, Division of Neurosurgery, BC Children’s Hospital, Vancouver, BC; ** UPMC Eye Center of UPMC, Pittsburgh, Pennsylvania; †† Department of Radiology, Children’s Hospital of Philadelphia.

## Abstract

**Background:**

We report the incidence of Chiari malformation I (CMI) in a cohort of 377 patients with isolated sagittal synostosis (ISS), which is to the best of our knowledge the largest such series reported to date.

**Methods:**

A retrospective review of patients seen at a single institution from 2007 to 2017 was completed. ISS, Chiari malformations (CMI and CMII) and hydrocephalus were diagnosed by a senior neuroradiologist (G.Z.). Patients who met the inclusion criteria were divided into early (group A) and late (group B) presenting groups, as well as operated (group I) and unoperated (group II) groups. The patients were further subdivided into group AI (early operated), group AII (early unoperated), group BI (late operated), and group BII (late unoperated). Once identified, patient notes were examined for the following data sets: date of birth, age of presentation, age at last follow-up, other systemic conditions as well as molecular testing results. Surgical interventions, ophthalmological, and other relevant data were recorded. Statistical analysis was run in the form of a chi-square test to identify a significant difference between each subgroup. A literature review of the incidence of Chiari malformations in patients with ISS was conducted.

**Results:**

Three hundred seventy-seven patients constitute the study’s total cohort (272 were males and 105 females). This cohort was divided into patients who underwent surgical repair of ISS (group 1: n = 200), and patients who did not (group 2: n = 177). The entire cohort was also divided into early (group A: n = 161) and late (group B: n = 216) presenting craniosynostosis. In the total cohort, 22/377 (5.8%) patients with CMI were identified. CMI was found in 14/200 (7.0%) patients in group I, and 8/177 (4.5%) patients in group II. CMI was found in 2/161 (1%) patients in group A, and 20/216 (9.2%) patients in group B. The incidence of CMI in group AI (early operated) was 2/151 (1.3%), in group AII (early unoperated) was 0/10, in group BI (late operated) was 11/49 (21%), and in group BII (late unoperated) was 9/167 (5.4%). Chi-square analysis revealed a significant difference between the incidence of CMI in the early-presenting (group A) and late-presenting (group B) groups (*P* = 0.001) and between the late-presenting operated (BI) and late-presenting unoperated (BII) groups (*P* = 0.001). The incidence of hydrocephalus was 1.6% (6/377) in the total cohort. However, all patients diagnosed with hydrocephalus came from group II (no surgical ISS correction). The incidence of hydrocephalus in group II was 3.3% (6/177). The incidence of hydrocephalus in group BII (late unoperated ISS) was 3.0% (5/167). The incidence of hydrocephalus in group AII (early unoperated ISS) was 9.0% (1/11).

**Conclusions:**

We noted the highest incidence of CMI—21%—in group BI (late-presenting operated). We noted hydrocephalus in group II (nonoperated), with the highest incidence of hydrocephalus found in the group BII (late-presenting unoperated) subgroup. We therefore recommend patients with ISS receive funduscopic examination to screen for raised intracranial pressure (ICP) associated with CMI and hydrocephalus, especially patients with late-presenting ISS.

## INTRODUCTION

Craniosynostosis and Chiari malformations are congenital anomalies that have varied etiologies.^1,2^ Craniosynostosis is characterized by the premature fusion of the cranial sutures. It occurs in 1 out of every 2,500 live births^3^ and may involve the coronal, lambdoid, metopic, and sagittal sutures. The gold standard for diagnosis is a 3D computed tomography (CT) scan.^4^ Sagittal craniosynostosis is the premature fusion of the sagittal cranial suture, resulting in a lengthened cranium in the anterior-posterior direction with stunted lateral expansion of the parietal and temporal regions. This restriction of growth results in a ridged sagittal suture, scaphocephaly and heightened frontal and occipital bones.^5,6^ Isolated sagittal synostosis (ISS) accounts for half of all cases of craniosynostosis, including syndromic and nonsyndromic cases.^7,8^ The premature fusion of the sagittal suture may be complete or involve either the anterior, middle, or posterior regions.^3,8–10^ The main indicator for surgical correction of ISS is to prevent the sequelae associated with raised intracranial pressure (ICP), which may be observed in 15–40% of cases, and provides a strong indication for surgery.^11–14^

Chiari malformation I (CMI) is defined as the herniation of the cerebellar tonsils through the foramen magnum into the spinal canal.^15^ A diagnosis of CMI is made by observing the tips of the cerebellar tonsils at least 5 mm below the foramen magnum. The gold standard for CMI diagnosis is MRI, but diagnosis can be made via CT scan. CMI is caused by crowding of the hindbrain due to decreased posterior fossa volume and is considered a deformation of normal cerebellar tissue. It may be congenital or present later in both children and adults. CMI can be a progressive condition, but because it is only visible on radiological examination—and is often asymptomatic—patients generally receive treatment if they are experiencing symptoms such as suboccipital headaches and tingling pains, or exhibit a syrinx on cervical MRI.^16–21^ It is controversial whether CMI occurs secondary to reduced intracranial volume.^22,23^ Although some cases of CMI with craniosynostosis may be independent congenital anomalies, many may be due to changes secondary to craniosynostosis, such as raised ICP secondary to intracranial crowding.^18^ The finding of raised ICP in at least 15–20% of single suture synostosis patients supports the idea that the etiology of CMI in patients with craniosynostosis might be distinct from patients without craniosynostosis.^11,13,14,17,24^ Studies have demonstrated that patients with complex and/or syndromic craniosynostosis diagnosis such as Crouzon’s, have CMI at rates between 50% and 100%,^25–29^ and have shown an association between certain nonsyndromic craniosynostosis types, especially lambdoid synostosis, and CMI.^30^

A type II Chiari malformation (CMII) involves herniation of the cerebellar vermis, tonsils, and brainstem tissue and is therefore much more severe than type I. Due to the formation of a myelomeningocele, CMII is typically symptomatic. Type III Chiari malformation (CMIII) is the most severe, and rare, form of Chiari malformation, occurring in association with a cervical encephalocele and often results in severe neurological defects. In CMIII, the brainstem, cerebellum, and matter from the fourth ventricle are often herniated into the encephalocele.^20^ Thus far, there have been case reports with small numbers describing patients with ISS and CMI.^31–33^ To the best of our knowledge, this is the largest retrospective study to date examining the incidence of CMI in patients diagnosed with ISS.

## METHODS

A retrospective review of patients diagnosed with only ISS was completed. Patients were identified through evaluation of institutional databases for the years 2007–2017. Once identified, patients’ charts were examined for the following data sets: date of birth, age of presentation, age at last follow-up, other congenital anomalies, other systemic conditions, and presence of hydrocephalus. Genetic testing was completed in 75 patients (20%). ISS, Chiari malformations (CMI and CMII), and hydrocephalus were diagnosed by a senior neuroradiologist (G.Z.). Each diagnosis of ISS was made via CT, and each diagnosis of CM was made via magnetic resonance imaging (MRI). Surgical indications for early and late-presenting patients with sagittal craniosynsotosis include papilledema, raised ICP and scaphocephaly. Additional measures include behavioral changes and performance on visual evoked potentials (VEPs).

Neuroimaging results of patients diagnosed with ISS and CMI were re-evaluated by an experienced pediatric neuroradiologist to confirm the original diagnosis. The patients were divided into the following groups: group I: patients with surgically corrected ISS, group II: patients with uncorrected ISS, group A: early-presenting patients (synostosis diagnosed at or before one year of age), and group B: late-presenting patients. These groups were further subdivided into AI: early operated, AII: early unoperated, BI: late operated, and BII: late unoperated. A chi-square test was used to determine if the differences in the incidence of CMI differed significantly between any of the 4 subgroups.

## RESULTS

The research team identified 400 patients with a diagnosis of ISS. Twenty-three of 400 (5.7%) patients who developed ISS secondary to surgical treatment (usually ventriculoperitoneal shunting) of hydrocephalus. These patients were excluded from analysis. Still, they had a high percentage of Chiari (type I and II). Of the 23 cases in our series that developed ISS after shunting, 2 (9%) had CMI, 4 (17%) had CMII, and 17 (74%) had no CM diagnosis.

The total cohort formally examined was composed of 377 patients (272 males and 105 females; Table 1). The mean age of CT diagnosis for the entire ISS cohort was 4.26 years (SD, 4.97 years; median, 2.13 years; mode, 0.19 years). Two hundred of 377 (53%) patients underwent surgical repair (group I), and 161 of 377 (42%) were diagnosed before 1 year of age (group A). Group I comprised 157 boys and 43 girls. The mean age of CT diagnosis for patients in group I was 1.37 years (SD, 2.72 years; median, 0.32 years; mode, 0.19 years). Group II (patients who did not undergo surgical ISS correction) comprised 120 boys and 57 girls. The mean age of CT diagnosis in group II was 7.40 years (SD, 4.99 years; median, 6.99 years; mode, 1.94 years). Group A (early-presenting ISS) comprised 118 boys and 43 girls. The mean age of CT diagnosis in group A was 0.30 years (SD, 0.23 years; median, 0.25 years; mode, 0.19 years). Group B (late-presenting ISS) comprised 154 boys and 62 girls. The mean age of CT diagnosis of patients in group B was 7.42 years (SD, 4.84 years; median, 6.25 years; mode, 1.94 years).

**Table 1.**
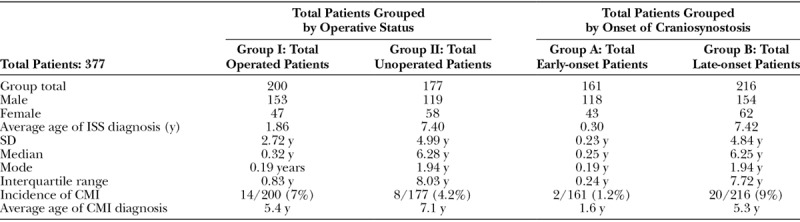
Incidence of CMI in ISS Early- and Late-onset Groups

In the total cohort, 22 of 377 (5.8%) patients with CMI were identified: 19 were male and 3 female. The average cerebellar tonsil descent for CMI patients was 8 mm. The average age of CMI diagnosis was 5.4 years (SD, 4.3 years). Fifty-nine percentage (13 of 22) patients diagnosed with CMI did not show any symptoms of the malformation, and 4 of 22 (18%) of these patients were diagnosed with a syrinx. Within the subgroups, the incidence of CMI was 7.0% (14 of 200) in group I (operated), 4.5% (8 of 177) in group II (unoperated), 1.2% (2 of 161) in group A (early-presenting), and 9.2% (20 of 216) in group B (late-presenting; Table 1). Within the further divided subgroups, the incidence of CMI was 1.3% (2 of 151) in group AI (early-presenting operated), 0% (0/10) patients in group AII (early-presenting), 21% (11 of 49) in group BI (late-presenting operated), and 5.4% (9 of 167) in group BII (late-presenting unoperated; Table 2). Chi-square analysis revealed a significant difference between the early (group A) and late (group B) presenting groups (*P* = 0.001) and between the late-presenting operated (BI) and late-presenting unoperated (BII) groups (*P* = 0.001). There was no significant difference found between the operated and unoperated groups (groups I and II), or between the early operated/unoperated groups (AI and AII). However, group AII contains 10 patients and is likely not powered enough to draw any conclusions. In the total cohort, 27% (6 of 22) patients with CMI underwent surgical repair for CMI, whereas 9.1% (4 of 22) underwent surgical repair of both their Chiari malformations and ISS. Three patients in group I and 3 patients in group II underwent CMI repair. Two patients in group I underwent simultaneous repair of ISS and CMI. 1 patient in group A (early-presenting ISS) underwent CMI repair, and 5 patients in group B (late-presenting ISS) underwent CMI repair.

**Table 2.**
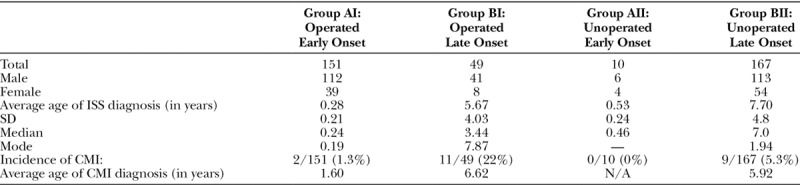
Incidence of CMI in ISS Cohort-subgroups

Nineteen of 22 (86%) patients diagnosed with CMI were diagnosed via MRI. The remaining 3 (all patients with late-presenting synostosis) were diagnosed via CT and were already in the care of plastic/neurosurgery for treatment other than CMI symptoms. Fourteen of 22 (64%) patients diagnosed with CMI and sagittal synostosis underwent repair of their sagittal synostosis. Of these, 10 underwent cranioplasty only and 4 underwent combined cranioplasty and decompression surgeries. All of these patients were late presenting and underwent the following operations: parietooccipital or suboccipital craniectomies with laminectomy for treatment of CMI, and calvarial expansion for repair of sagittal synostosis.

Postoperative MRI imaging of patients who underwent sagittal synosostosis repair, without CMI decompression, was completed in 9 patients. Of these, 5 patients had CMI that were stable, and 4 demonstrated improvement of cerebellar tonsil herniation. These patients who improved underwent a mix of surgical interventions: specifically total (with and without Pi hung span modification) middle and anterior half cranial vault remodeling procedures.

The incidence of hydrocephalus was 1.6% (6/377) in the total cohort. However, all patients diagnosed with hydrocephalus came from group II (no surgical ISS correction). The incidence of hydrocephalus in group II was 3.3% (6/177). The incidence of hydrocephalus in group BII (late unoperated ISS) was 3.0% (5/167). The incidence of hydrocephalus in group AII (early unoperated ISS) was 9.0% (1/10). Only 222 of 377 patients (58%) received an ophthalmic evaluation. However, of the patients with CMI, we noted 18 of 22 patients (82%) received an ophthalmic examination, and of these, 5 of 22 (23%) patients had papilledema on examination. One of these patients had strabismus.

## DISCUSSION

To the best of our knowledge, this is the largest study of its kind to date. It includes operated and unoperated patients and distinguishes between early and late-presenting ISS, which we feel allows us to better examine the natural history of ISS. Previous literature investigating the incidence of CMI includes operated patients only and reports an incidence varying from 0% to 8%.^24,34–35^ Our data demonstrate a similar incidence for the same population (group I) of 6.5%. The incidence of CMI in certain craniosynostosis varieties is well known in patients with syndromic, multisutural, and single-suture lambdoid synostosis types. The latter is most closely associated with CMI, relative to all other single suture craniosynostosis varieties.^24,26,28–30,33,35,36^ We sought to understand the extent of correlation between ISS and CMI and found that over 20% of late-presenting sagittal synostosis patients who underwent surgery had CMI. Although previously published,^25,33^ we believe the association between ISS and CMI malformations has been underreported mainly due to the nature of smaller retrospective studies and the nonsegregation of early versus late-presenting groups. We only found patients diagnosed with hydrocephalus in group II, which suggests that patients with unoperated ISS are more likely to develop hydrocephalus. Although it is possible that some of the patients with hydrocephalus and ISS had other systemic issues, all patients presented to craniofacial or neurosurgery with sagittal synostosis as their chief complaint. In this cohort, 50% of all patients had a systemic or syndromic diagnosis, but by far the majority were diagnosed after craniosynostosis (as yet unpublished). Unfortunately, only 222 of 377 (58%) of our patients had a baseline ophthalmic observation. However, the incidence of CMI and hydrocephalus in the unoperated and late-presenting patient groups strongly suggests that all patients with ISS should have a baseline ophthalmic evaluation, since papilledema may reflect raised ICP related to CMI or hydrocephalus. We note that since this project began, the protocol has shifted to ensure all patients with ISS receive ophthalmic evaluation.

Our patient cohort includes older pediatric patients, as well as all radiologically diagnosed cases of ISS seen during the 2007–2017 interval. At the time not all patients—particularly those who came in for trauma were seen by ophthalmology—as well as many patients for whom surgery was not recommended, but who were scaphocephalic and asymptomatic. The mean age of diagnosis for craniosynostosis patients in our study was 4.26 years. This is older than most previous studies.^24,34^ CMI is a progressive condition, so physicians can expect the malformation and its symptoms to worsen over time,^26^ which is supported by our data. Specifically, within our cohort, 40% (9/22) of patients with CMI were symptomatic and/or were diagnosed with a syrinx. Additionally, those 7 of 11 (64%) patients with late-presenting sagittal synostosis and CMI who underwent surgical correction of their synostosis were symptomatic for CMI. Subsequently, having an older cohort of patients might explain the high incidence of CMI (and *symptomatic* CMI), as a child with symptomatic CMI is more likely to undergo diagnostic imaging and surgical intervention than an asymptomatic case.

Though they were analyzed separately, we identified 23 of 400 (6%) patients who developed ISS secondary to treatment for hydrocephalus. The etiology of secondary craniosynostosis is controversial,^36–41^ and though thought to be rare, the incidence of secondary synostosis has been reported at as high as 12%.^36^ Secondary craniosynostosis is known to occur in patients with multisutural as well as single suture craniosynostosis.^37,39,42,46^ Of the 23 cases in our series that developed ISS after shunting, 2 had CMI, 4 had CMII, and 17 had no CM diagnosis. One report describing the effect of CSF shunting on the brain describes a single patient with hydrocephalus and CMII, but the patient did not have ISS.^47^ We have been unable to find any cases of CMII in secondary ISS or—more generally—secondary craniosynostosis patients, and thus CMII in patients with secondary ISS may be a novel finding. There have been no large-scale studies investigating the role of genetics in cases of secondary synostosis, and though we had a relatively small number of these patients, we noted prematurity in 26% (6/23), as well as 15q13.3. and 16p.13.3 single copy deletions. We also noted 30% (7/23) experienced seizures in this patient group, and a single patient had clubfeet. The relevance of this unclear but is being further investigated by our group.

## CONCLUSIONS

Previous studies investigating the incidence of CMI in craniosynostosis patients have examined predominantly operated patients and have not made a distinction between early- and late-presenting groups. In our cohort, the incidence of CMI in patients who underwent surgical correction for ISS—without prior surgical intervention for hydrocephalus—was 7.0% (14/200), but when subdivided into early-presenting operated (AI) and late-presenting operated (BI) groups, the incidence of CMI is 1.3% and 21%, respectively. To the best of our knowledge, the incidence in the late-presenting operated group (BI) is the highest incidence of CMI in ISS patients recorded to date. Additionally, we noted higher incidences of hydrocephalus in the nonoperated, and late-presenting ISS groups. We therefore recommend patients with ISS receive funduscopic examination to screen for raised ICP associated with CMI and hydrocephalus, especially in children who present with late-presenting ISS (diagnosed after a year of age).
